# The role of the prefrontal cortex in social interactions of animal models and the implications for autism spectrum disorder

**DOI:** 10.3389/fpsyt.2023.1205199

**Published:** 2023-06-20

**Authors:** Alok Nath Mohapatra, Shlomo Wagner

**Affiliations:** Sagol Department of Neurobiology, Faculty of Natural Sciences, University of Haifa, Haifa, Israel

**Keywords:** autism, prefrontal cortex, rodent models, social interaction, social behavior

## Abstract

Social interaction is a complex behavior which requires the individual to integrate various internal processes, such as social motivation, social recognition, salience, reward, and emotional state, as well as external cues informing the individual of others’ behavior, emotional state and social rank. This complex phenotype is susceptible to disruption in humans affected by neurodevelopmental and psychiatric disorders, including autism spectrum disorder (ASD). Multiple pieces of convergent evidence collected from studies of humans and rodents suggest that the prefrontal cortex (PFC) plays a pivotal role in social interactions, serving as a hub for motivation, affiliation, empathy, and social hierarchy. Indeed, disruption of the PFC circuitry results in social behavior deficits symptomatic of ASD. Here, we review this evidence and describe various ethologically relevant social behavior tasks which could be employed with rodent models to study the role of the PFC in social interactions. We also discuss the evidence linking the PFC to pathologies associated with ASD. Finally, we address specific questions regarding mechanisms employed by the PFC circuitry that may result in atypical social interactions in rodent models, which future studies should address.

## Introduction

The prefrontal cortex (PFC) is critical for various aspects of mammalian social behavior, including social motivation, recognition, and decision-making (1–3). In humans, the medial PFC (mPFC) is involved in high-order aspects of social interaction, such as self-referential processing, mentalizing, and emotional regulation (4–6). At the same time, deficits in PFC function have been implicated in various neuropsychiatric disorders, including autism spectrum disorder (ASD). Individuals with ASD exhibit atypical social behavior and deficits in social cognition, such as an impaired theory of mind and a lack of social interest (7, 8). Neuroimaging studies have revealed altered PFC activity in individuals with ASD during social tasks (9, 10). As such, understanding the molecular, cellular, and network mechanisms underlying the role of the PFC in social behavior and its dysfunction in ASD may be critical for developing effective treatments for individuals diagnosed with this disorder.

Research using animal models has provided significant insight into the neural circuitry underlying social behavior, including the role of the PFC in social interactions (11–14). Anatomically, the PFC is a complex brain structure with multiple sub-regions, each with a distinct function and connectivity pattern (15, 16). In rodents, most studies have focused on the mPFC, including the prelimbic and infralimbic regions and their downstream projections to the striatum (17), amygdala (18), hypothalamus (19), hippocampus (20) and brainstem (21). These sub-regions were shown to be involved in various aspects of social behavior, including social recognition (22), social approach (23), and aggression (24, 25). Moreover, studies have demonstrated that rodents exhibit complex social behaviors, including social hierarchy (26), empathy (27), and territoriality (28), making them a valuable model for studying the biological mechanisms underlying mammalian social behavior. Accordingly, multiple behavioral tasks have been developed to assess rodent social behavior and the role of the PFC therein, including the three-chamber, social recognition, social habitation/dishabituation, and resident-intruder tests (29–32). Such studies have shown that mPFC lesions or manipulations can lead to deficits in social behavior in rodents (33, 34).

Here, we provide an overview of the role of the PFC in the social behavior of animal models and the implications for understanding possible mechanisms underlying social deficits in ASD. The review discusses anatomical and functional homologies of the PFC in rodents and humans, and its role in various aspects of social interactions. Additionally, current literature on PFC involvement in social behavior deficits that lead to ASD symptoms is highlighted.

## Social interactions involve social motivation, recognition, and decision-making

Social interactions involve complex information-processing tasks that can broadly be defined as detecting and interpreting social cues and responding appropriately to evolving social contexts (3). By nature, social interactions are multi-faceted and require the integration of external multi-modal sensory information with internal processes. Here, we aim to focus on the following aspects of the process: (1) the motivation for social interaction, which is an internal process; (2) emotional/empathic reactions in response to social cues; and (3) group dynamics, which involve mutual relationship between the subject and others (4, 35–37). These aspects are not mutually exclusive (38) and together affect behavioral decisions. This is exemplified by going out to dinner at a restaurant. This involves interactions with the staff, the degree to which heavily relies on the internal motivation of the subject to interact. The subject’s satisfaction with the food and the staff performance, as well as the subject’s perception of their emotions. Will lead the subject to either compliment or complain about the staff. Moreover, verbal and emotional communication between the dining partners during dinner will depend on whether the environment is friendly or professional. Thus, social motivation, emotional perception of self and others, group dynamics, and the social context all integrate to determine social behavior.

Social motivation, or the willingness to pursue social interactions, is a fundamental aspect of the decision-making process in a social context. Such motivation and subsequent rewarding experiences require the subject to approach social partners and engage them (35). Accordingly, approaching a conspecific is a highly conserved phenotype in multiple species (38, 39). This aspect of social behavior and cognition emerges early in development, with young infants tending to recognize and initiate interactions with their parents (40, 41). Infants must thus recognize familiar faces for proper decision-making in their social contexts from a very early age (42, 43). Hence, social motivation serves as the developmental and evolutionary foundation for complex social behaviors.

The ability to interpret others’ intentions and mental states heavily governs social interactions in any social context. Emotional comprehension, like evaluating social motivation, recognizing body language and facial cues, as well as interpreting implicit and explicit biases of others, are essential to any social interaction. This social cognition process, termed “theory of mind” (44, 45), heavily influences individual social decision-making (46).

Social interactions require effective group dynamics, allowing individuals to develop healthy and essential group relationships (47). Hierarchical, territorial, cooperative, and interdependent social behavior are observed in multiple species. Studies have highlighted the role of social hierarchy in individual well-being, leading to better availability of resources essential to survival, such as food, space, and mating partners (48, 49). Investing in a territorial or hierarchical structure is also an essential decision-making process in which individuals gauge their metabolic energy before involving themselves in conflicts related to group social structure (48). Moreover, the social context of a conflict weighs heavily on an individual’s role in the group dynamics, with an effective change in this role relying on a correct decision-making process.

Social decision-making involves multi-faceted processes, Thus, multiple malfunctions can lead to the atypical social behavior characterizing multiple neuropsychiatric disorders, such as autism spectrum disorder (ASD). Impaired recognition of familiar faces or reduced motivation for social interactions have been reported in ASD (7, 50). Indeed, infants lacking social interest are likely to develop social cognition deficits (51), such as the impaired theory of mind (52, 53). Maladaptive social decision-making capabilities are prevalent in ASD and serve as predictors of overall mortality due to the effects of poor interpersonal relationships on mental and physical health (54). Neuroimaging studies subsequently revealed the involvement of many interconnected brain regions during social decision-making (55). Assigning the process to functionally relevant brain entities is critical for explaining their roles in the atypical behaviors exhibited by individuals diagnosed with ASD.

## Evidence for the role of the PFC in human social interactions

The PFC has been linked to various aspects of cognition and behavior, such as working memory, decision-making, goal-directed conduct, and social behavior (32, 56, 57). The PFC presents significant yet variable connections to both cortical and sub-cortical areas of the brain, including the hippocampus, amygdala, hypothalamus, and nucleus accumbens, as well as areas associated with sensory-motor functions (18, 58). Many of these areas were shown to be involved in social decision-making (59, 60). Thus, the PFC contributes to all aspects of social interactions, in collaboration with other cortical and sub-cortical regions.

Various regions of the PFC also process distinct aspects of social information (57, 61). Regions that process social motivation play inherent roles in reward, valence, and affiliation and include the orbitofrontal and perigenual anterior cingulate cortices (ventro-medial prefrontal cortex; vmPFC: BA 10,11,12, 25, and 32; orbito-frontal cortex; OFC: BA 10 and 11; and anterior cingulate cortex; ACC: BA 25 and 32) (61). Multiple studies have reported a role for the vmPFC in social motivation and reward. Humans with vmPFC lesions exhibit impairments in emotional recognition and making moral decisions (62). They also failed to learn from recent reward history in a pro-social game (63). Other studies concluded that the OFC plays a role in decision-making based on the valence of the stimuli (64, 65). Additionally, the vmPFC is active when subjects feel socially accepted and comprehend rewarding social cues (66). Interestingly, specific impairments in the tendency of ASD patients to find social stimuli incentivizing or motivating are similar to those seen in humans with vmPFC lesions (67).

Social interactions that necessitate knowledge of oneself and others are consistently associated with activation within the PFC (specifically, the medial and dorso-medial prefrontal cortex; dmPFC). The mPFC is effectively activated while comprehending self-bias and those of others (in line with the theory of mind), beliefs, moral decisions, and emotional states while empathizing with others’ pain and during cooperation [(68, 4);]. Functional magnetic resonance imaging (fMRI) studies showed this region to be active during cooperative tasks among humans, tasks in which ASD patients perform poorly due to lower attention to social cues (69–71). Evidence of decreased activity and connectivity in the mPFC of ASD patients has been reported and are likely to significantly contribute to the social and behavioral deficits presented by these individuals. Studies also demonstrated that ASD patients lack adaptive control in comprehending and adapting their behavior according to an unfair social context or their partner’s emotional expressions (72, 73). Moreover, the infant mPFC is responsive to social cues, like a parent’s face and gaze (74). Furthermore, in contrast to patients who sustained damage to their mPFC as adults, patients who sustained damage to this region as children demonstrate anti-social behavior and poor moral decision-making in adulthood (62). Together, these studies point to the mPFC as serving a crucial role in the forming of proper social cognition in humans from early development stages.

There have been attempts to define the role of distinct PFC sub-regions in separating internal from external social reasoning. The mPFC has been reported to be involved in tasks that involve processing of internal states of self and others, such as empathy, self-reflection, and vicarious moral reasoning (75–77). In contrast, the lateral PFC (lPFC) is part of a network activated by externally guided information processing in the social domain, such as imitation, abstract social reasoning, and internal conflict resolution (78, 79). In addition to ASD, there is strong evidence that patients with other neuropsychiatric disorders, like schizophrenia (SCZ), display hypo-activity in the dorsal lPFC during social interactions (80, 81). Recent works using transcranial direct current stimulation with SCZ and ASD patients described improved social and emotional behavior (82, 83). Yet, despite the apparent improvement in patient behavior following treatment, there was a lack of mechanistic links and specific definitions of such interventions for comorbidities like depression and anxiety. Thus, the particular sections of the PFC that implicitly and explicitly affect individual emotional comprehension remain elusive, although solid evidence points toward the mPFC and lPFC.

The third aspect of social interaction, group dynamics, combines social motivation and emotional comprehension of the social context. fMRI studies found neural correlates of social hierarchy and group dynamics to occur in the PFC (84, 85), as well as in sub-cortical regions, like the amygdala and ventral striatum, that demarcate distress from rewarding social experiences (86, 87). lPFC bias to the superior as opposed to the inferior player in a monetary reward task was only observed in a social context, i.e., with other players, implying that involvement of the lPFC in processing hierarchical information is specifically social in nature (88, 89). Patients with dorsal and lateral PFC lesions do not understand changes in social hierarchy and fail to learn them (67, 90). Thus, activity in the PFC and sub-cortical regions coordinates proper behavioral responses when the social hierarchy is changing, with such knowledge having to be constantly updated in these regions.

**Figure 1:**
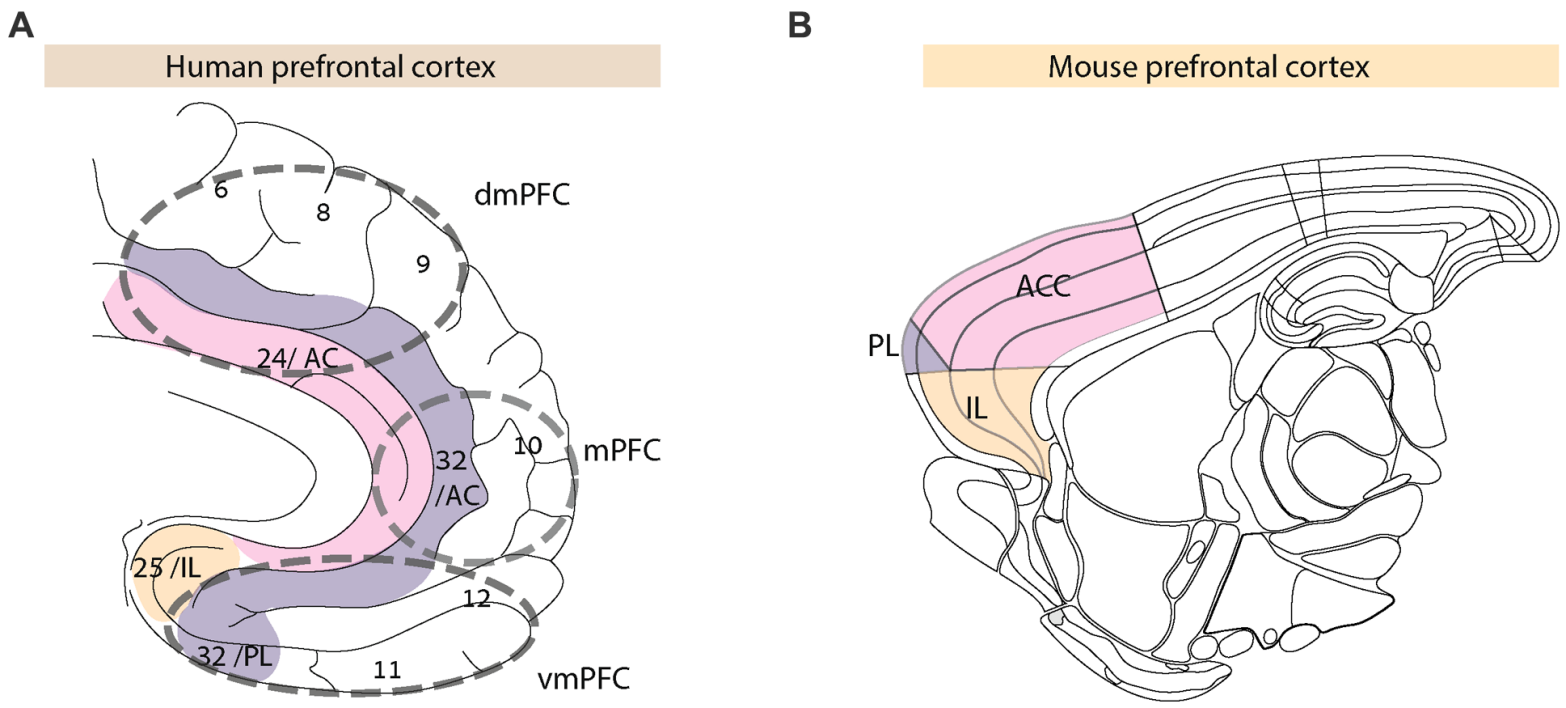
Anatomy of prefrontal cortex in human and mouse the human prefrontal cortex **(A)** includes Broca areas (BA) 6, 8, 9, and 24 (comprising the anterior cingulate, AC), 10, 11, 12, 25, and 32. The colored regions define PFC sub-divisions in humans and the corresponding homologous regions in mice (91–93) **(B)**.

In summary, the PFC and its connections to sub-cortical brain regions regulate and encode various aspects of human social interactions (Figure 1). PFC sub-divisions contribute to social motivation, reward, cooperation, and mentalizing of self and others’ socio-emotional states. In the following sections, we compare the above evidence supporting the role for the PFC in human social interactions with what occurs in rodents.

## Social interactions in an animal model: practical tools for studying ASD social deficits

Non-human primates (NHPs) present rich social behaviors, such that studies on these models may directly inform on clinical interventions for neuropsychiatric disorders such as ASD. Relevant studies are, however, restricted by small sample size, lack of effective circuit-specific manipulation tools, and the general difficulty and slowness of experimentation. Furthermore, limitations in specific genetic lines that mimic mutations found in ASD patients hinder efforts aimed at mechanistic understanding of modifications in NHP social interactions. At the same time, rodent models represent effective and valuable systems for addressing specific questions regarding biological mechanisms and brain circuits involved in social behavior and their alterations by ASD-associated genetic mutations.

From rodents to primates and humans, social interactions and their underlying neural processes have been remarkably conserved. Nevertheless, the neurobiological mechanisms and brain circuits contributing to rodent social interactions remain elusive and have been only partially explained to date. The following section highlights the anatomical correlates of rodent social interactions that align with the human PFC.

### Anatomy of the rodent PFC

Historically, anatomical similarities between the human and rodent PFC gave rise to multiple controversies (91, 93–96). Studies of functional correlates indicated the rodent PFC as being involved in non-social behavior, like working memory (97), impulse control (98), attention, and goal-directed behavior (99, 100). The rodent prelimbic cortex (Figure 1) seems homologous to human BA 32 that is part of the dorsal and ventral PFC, including the lateral PFC (96, 101). At the same time, the rodent infralimbic cortex is considered to be homologous to BA 25, a part of the ventromedial PFC in humans. The rodent medial OFC and ACC share homologies with the human OFC and dorsomedial PFC, respectively. The granular cortical structure of the rodent PFC does not entirely match its human counterpart [see (93) for detailed comparisons]. Unlike the human PFC, the rodent PFC receives and projects extensively to other cortical and sub-cortical brain regions, specifically, limbic and midline thalamic regions that densely innervate the PFC.

Previous efforts indicated the existence of a dichotomy between sub-regions of the rodent PFC in processing social interactions (102, 103). Because of this, it is crucial to employ behavioral paradigms that are ethologically appropriate and take advantage of typical rodent actions that are involved in social interactions. In the following section, we discuss how the rodent PFC regulates social interactions in ethologically relevant tasks of social behavior and how specific pre-clinical models of ASD may highlight the role of the PFC in such pathologies. We also review the extensive literature on rodent PFC involvement in numerous social behavior tasks by concentrating on three distinct aspects of social interactions and on studies that specifically explore these aspects.

## The role of the PFC in rodent social interactions

Multiple tasks have been developed to gauge rodent social motivation (31). It should be noted that the parameters quantified in these tasks, such as the time spent near social stimuli, reflect traits that are vastly different from those humans employ during social interactions (104, 105). Furthermore, rodents predominantly utilize the olfactory sensory system during social interactions (106), in contrast to predominant dependence of human social interactions on visual and auditory cues (107).

### Tasks that assess social motivation and recognition

Multiple tasks have been developed to assess the recognition of conspecifics (social recognition) and the motivation to orient and approach them (see (32) for a detailed list of behavior tasks used to test rodents). The earliest social recognition task, the social habituation/dishabituation test, relied on a series of encounters with the same conspecific (social stimulus) and finally, with an unfamiliar one (108). Such assays reveal that in general, subjects gradually lose the motivation to interact when encountering the same (familiar) social stimulus in subsequent trials (the habituation phase), indicative of recognition of the familiar stimulus. A subject’s interaction time returns to the level of the first trial when exposed to an unfamiliar stimulus (dishabituation), thus controlling for changes in general social motivation. This task effectively reports on short-term and long-term memory in rodents, despite exposing confounds of internal state and novelty that cannot be controlled (109).

Social discrimination tasks were devised to probe the motivation to interact with specific stimuli while using appropriate controls that account for the novelty of a stimulus. For example, the social novelty preference task considers the time spent investigating (i.e., sniffing) a novel stimulus as opposed to a familiar cage-mate or a recently encountered conspecific to control for aggression due to male pheromones and general social motivation (110). These discrimination tasks provide information on different behavioral dynamics (111), which cannot be analyzed in the habituation/dishabituation task. Another variation of social recognition task specifically designed for a monogamous species of voles is the partner preference test. These monogamous rodents preferably interact with their partner after pair-bonding, relative to a stranger (112).

### Tasks that test affective/emotional behavior

**Figure 2:**
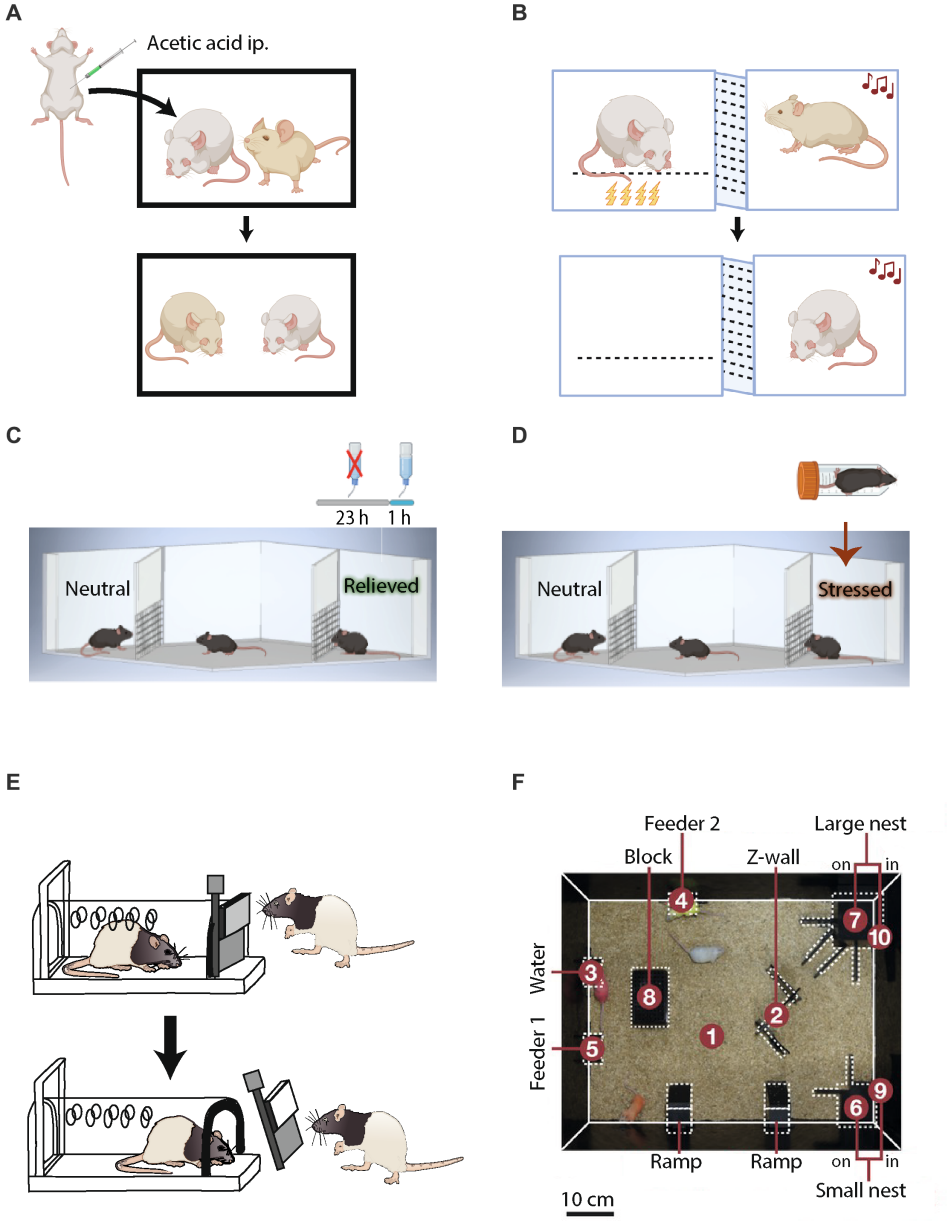
Tasks to test affective emotional, empathic and group dynamics in rodents. **(A)** Social transfer of fear in a rodent observing a demonstrator in pain due to acetic acid injection. **(B)** Social transfer of fear in a rodent observing a demonstrator experiencing foot-shock-induced pain. **(C)** Affective emotional state preference of conspecifics experiencing positive emotions, such as relief from thirst or **(D)** negative emotions, such as stress due to being restrained for a while, over neutral conspecifics. **(E)** Pro-social empathic behavior of rats freeing a captive conspecific. **(F)** Semi-natural social box for studying rodent group dynamics.

Some of the earliest proof of emotional cognition appears in works where rats (i.e., observers), trained to receive food rewards in lever press tasks, reduced the amount of lever pressing as they observed another rat (i.e., a demonstrator) being exposed to foot shocks. The study reflected the transmission of emotional state between observer and demonstrator rats (113). Similarly, mice and rats demonstrated the social transmission of pain and analgesia (114, 115), fear (116, 117) (Figure 2A), and food preference (118).

Several tasks indicate that rodents display emotions, specifically fear, and thus enable social transmission of emotional information. Rodents, moreover, respond to emotional states of other individuals. In transfer learning procedures, such as fear conditioning by proxy, a rat exposed to a novel tone while in the presence of a cage-mate who was previously fear-conditioned to that tone will freeze (119) (Figure 2B). In another procedure, known as social harm aversion, rats avoid a specific task (like lever pressing) if it causes harm to others (120). This behavior is affected by the outcome. For instance, positive outcome behavior occurs more often than does a decrease in negative outcome-related behavior (121).

Recent studies have tested the capability of rodents to recognize and discriminate emotional states of conspecifics (122). In the positive mode of a relevant task, which uses the setup of social discrimination tasks, one of two presented social stimuli is associated with deprivation of water in the home cage for the preceding 23 h and a quenching of thirst for an hour before the experiment. This manipulation of water availability in the home cage induces a “relieved” state in the social stimulus, drawing more attention from the subject than a control stimulus, which remains in neutral conditions (Figure 2C). On the other hand, the negative mode of this test probes discrimination of a negative emotional state, induced in a social stimulus by foot shocks or a short period in a restrainer, as compared to a neutral stimulus. In both positive and negative conditions, the subject mouse prefers interacting more with the arousing stimuli.

### Tasks that test empathic behavior

An behavioral task in which rats persistently try to free a captive conspecific, despite the temptation to instead consume a highly palatable food presented in the same arena, demonstrates empathy in these animals (123) (Figure 2D). Food-sharing tasks also reveal rats to be pro-social and empathic toward cage-mates. For example, Norway rats shared more palatable food with a partner who provided them with a piece of banana than with a partner who provided a less preferred piece of carrot (124). Rats also displayed pro-social behavior by providing food rewards to their cage-mates, even when they did not benefit from the decision to share food (125). In a consolation test of monogamous voles that quantifies the amount of allogrooming of a familiar, as compared to a stranger demonstrator, when the demonstrator was exposed to mild foot-shocks as stress, these rodents performed allogrooming of their stressed familiar partners so as to reduce their stress. The test thus differentiates empathic responses of a vicarious nature from general stress-coping behavior (126). In summary, these studies open ample avenues to study neural mechanisms of emotional recognition and empathy in rodents.

### Tasks that test group dynamics behavior

Social hierarchies emerge in mice when they live in densely populated conditions, where competition for territory, housing, mates, and food plays an essential role in the survival of the individual. Introducing pairs of cage-mates from opposing ends of a tube that does not allow sufficient space for a mouse to turn around or for both mice to pass each other offers one way to measure social dominance (127). Alternatively, semi-natural home cages (Figure 2E) that mimic large mouse colonies have been used to study dominance and hierarchical behavior (128, 129). Affective cooperation and altruistic behavior, investigated in rodents using lever pressing tasks, were shown to be influenced by the hierarchal stature of an animal in the group (130).

## What role does the rodent PFC play during social interactions?

Animal models support literature implicating the human PFC in social motivation, in conjunction with sub-cortical areas, such as the nucleus accumbens (NAc) and ventral tegmental area (VTA), which mediate the rewarding aspects of social interaction (131, 132). Although lesion studies have provided evidence for the crucial role of the PFC in social motivation (133, 134), such non-specific manipulation may damage nearby regions and axonal projections around the lesioned areas. Still, a comprehensive study examining murine whole-brain c-Fos expression in a social context revealed that social interaction strongly activates the mouse PFC (135).

**Figure 3:**
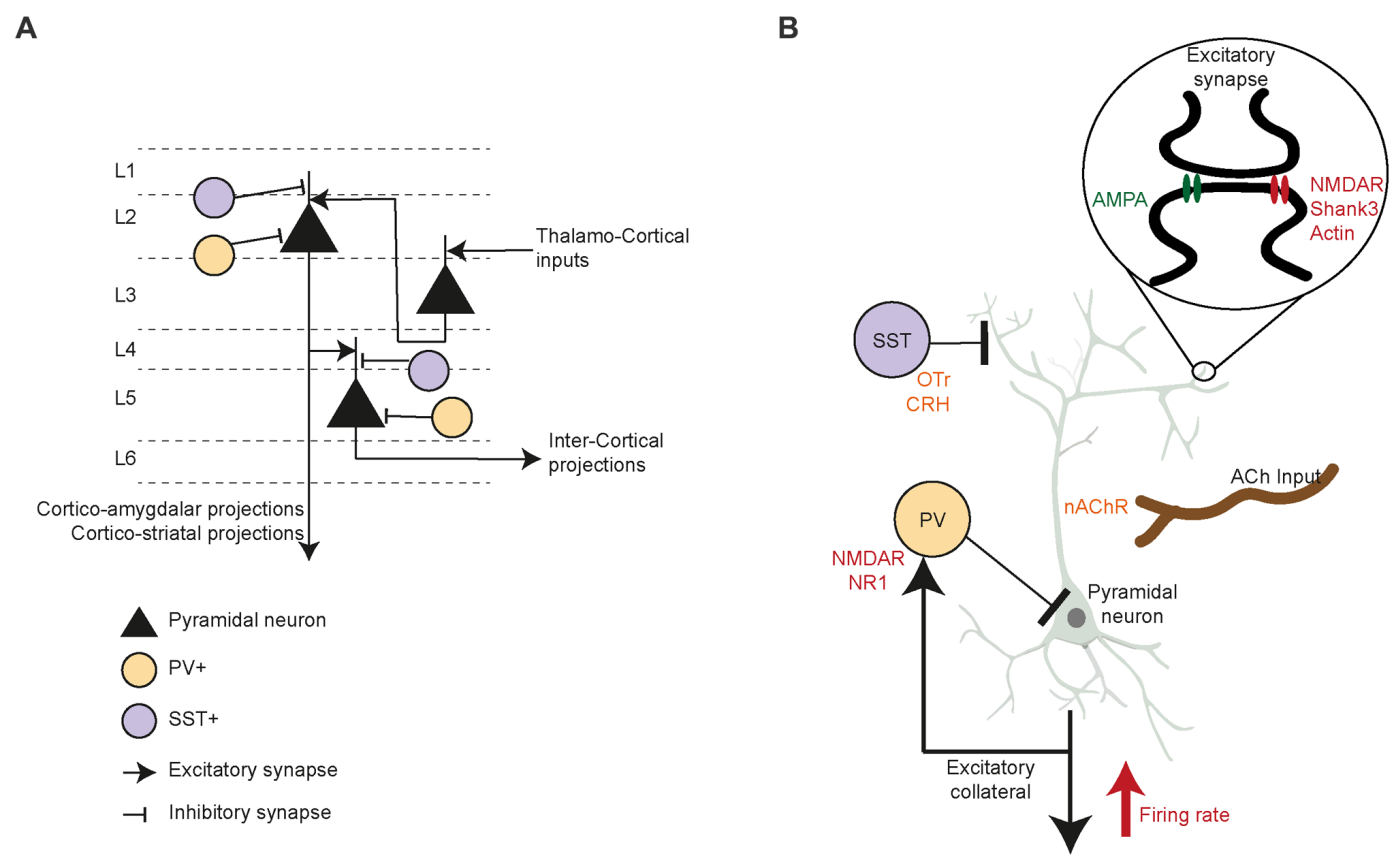
Prefrontal circuit’s specific components and their role in social interaction. **(A)** PFC circuitry and neuronal cell types driving inter and intra cortical excitatory drive. Specifically, the interneurons PV+ and SST+ inhibitory control over layer 2 (L2) as well as layer 5 (L5) pyramidal neurons. **(B)** Pyramidal neurons firing rate in PFC is modulated by PV+ and SST+ inhibition to the cell body and apical dendrites, respectively. Higher firing rate of the pyramidal neurons correspond to increased social motivation in mice. While the SST+ neurons are reported to be modulated through oxytocin and corticotrophin releasing hormone, specifically during social motivation and novelty preference behavior in rodents. While cholinergic projections into pyramidal neurons regulate social motivation and memory through nicotinic acetylcholine receptors. Further, low NMDA NR1 in PV+ neurons reduce social investigation. Excitatory synapses are specifically affected by structural protein Shank3 deficiency along with disruption actin formation, which cause low social motivation. Similarly, reduced excitatory post-synaptic currents due to low NMDA and AMPA receptors cause significant imbalance in prefrontal circuit E/I imbalance.

PFC circuitry is precisely arranged, presenting an array of interneurons that inhibit circuit activity, as well as neuromodulator inputs that rely on acetylcholine, dopamine and oxytocin. In mice, PFC circuitry is characterized by the canonical flow of excitation between cortical layers (Figure 3A), such as thalamo-recipient pyramidal neurons in layer 3 which send excitatory inputs to layer 2 pyramidal neurons. These layer 2 cells descend in turn to layer 5 pyramidal neurons (136). GABAergic interneurons (i.e., parvalbumin (PV+) and somatostatin (SST+) neurons) strongly control the excitatory drive of long-range and local intercortical-projecting pyramidal neurons in the PFC. These PFC interneurons display remarkable selectivity for connections with pyramidal neurons. In superficial layers, the PV+ and SST+ cells preferentially target layer 2 cortico-amygdalar and cortico-striatal pyramidal neurons (137, 138), whereas deeper in the cortex, the interneurons synapse solely with pyramidal neurons that target other pyramidal neurons (136, 139, 140). Many studies of pre-clinical animal models of ASD have reported decreased inhibitory neurotransmission in the PFC (141, 142), leading to low sociability, vocalization, and reciprocal social interactions (143). Excitatory/inhibitory (E/I) balance changes during development are linked to a critical period of plasticity in the PFC (144, 145). Post-mortem studies in ASD patients (146) extensively indicate reduced GABA receptors expression (147–149), increased Glutamatergic receptors expression (150, 151), and a low number of PV+ neurons in prefrontal cortex (152) which could result in the E/I imbalance. ASD patients show decreased gamma oscillation power, indicative of fast-spiking neurons firing at lower rates (153, 154). Studies in ASD patients showed higher numbers of dendritic spines, overall increased within-region connectivity, and a reduction in long-range connections of the PFC (155–157). Moreover, fMRI studies reported hypoactivation of the ACC to social reward in ASD compared to typically developing controls (158, 159), which indicate that these patient process socially rewarding and motivating cues abnormally (160). Therefore, investigating how alterations in the PFC circuitry affect social motivation and behavior may be essential for exposing the underlying mechanism of social deficits seen in ASD (161). Below, we further review the evidence that modified PFC circuitry interferes with social motivation.

Direct intervention in the E/I balance within the PFC circuitry profoundly affects the social motivation of adult mice. In seminal work, researchers optogenetically manipulated the neural activity of specific PFC neuronal populations during reciprocal interaction with juvenile conspecifics and in the three-chamber sociability task (162). Increasing excitatory activity by stimulating pyramidal neurons disrupted social exploration in the unrestricted interaction test and social preference in the three-chamber test. These deficits were brought down by activating inhibitory PV+ interneurons simultaneously with pyramidal cells, emphasizing the crucial role of an appropriate E/I ratio in the PFC for proper social motivation in mice.

### Pre-clinical models of E/I balance and its role in social motivation

Multiple synaptic or circuit-level factors establish and tightly regulate neuronal E/I balance (163). The balance between excitatory and inhibitory synapses in the brain is maintained through a complex interplay of several factors. These include the development and functioning of these synapses and the signaling pathways and mechanisms that regulate their plasticity. Homeostatic synaptic plasticity and intrinsic neuronal excitability also play roles in this delicate balance (164). At a higher level, E/I balance is regulated by the activity of different circuits, such as local circuits that involve distinct types of interneurons. These interneurons play a crucial role in regulating the activity of pyramidal neurons and modulating long-range connections (165, 166).

In the context of genetic risk factors for ASD, multiple studies have examined the E/I balance and its disruption in the PFC. Malfunctions of alpha-amino-3-hydroxy-5-methyl-4-isoxazole propionic acid (AMPA), N-methyl-D-aspartate (NMDA), and metabotropic glutamate receptors were found to affect the E/I balance in parallel to social behavior. For instance, Gandal et al. (167) showed that mice expressing low levels of the NMDA receptor *NR1* subunit in the PFC display low social motivation, decreased ultrasonic vocalizations, and abnormal gamma synchrony. Studies using genetic pre-clinical models linked reduced interneuronal markers in pre-frontal regions to imbalances in the E/I ratio due to a low level or lack of inhibitory control of pyramidal neuron excitability (168, 169). The maladaptive developmental trajectory of inhibitory interneurons and their role in later dysfunction of the PFC circuit have been widely studied (152, 170–172). While the impact of these deficits is global and affects multiple nodes of the social decision-making network that involves social motivation, the PFC is particularly susceptible. For instance, *Shank3*-deficient mice have been shown to lack social motivation and exhibit specific deficits in PFC circuitry, such as reduced NMDA-based excitatory post-synaptic currents (EPSCs) and a low number of F-actin filaments. These were rescued upon depolymerization of the actin filaments following systemic or focal treatment (173). Recent work involving circuit-specific mutation of *Shank3* in PFC-to-basolateral amygdala-projecting neurons recapitulated social motivation deficits and synaptic hypoactivity (174). In addition, chemogenetic activation of pyramidal neurons in the PFC of these mice rescued social interactions in the three-chamber task, as well as NMDA receptor-dependent EPSCs (175). Thus, PFC circuit dysfunction, especially of excitatory neurons projecting to the amygdala, directs social motivation deficits, at least in *Shank3*-deficient mice. However, mutations of the NMDA receptor *NR1* subunit in the PFC and hippocampus of adult mice did not decrease social novelty preference and sociability in the three-chamber task (176). Taken together, the development and early childhood susceptibility of interneurons may play a significant role in the PFC circuit and E/I balance abnormalities (Figure 3B) seen in ASD models (177, 178).

In addition to excitatory glutamatergic and inhibitory GABAergic activity, many neuromodulators alter PFC activity. Specific lesions of cholinergic projections into the PFC reduced rat social interactions in an open field arena (179). Distinct cholinergic inputs from the basal forebrain seemed to regulate different aspects of social interactions, namely social motivation and memory (180). Moreover, cholinergic signaling through nicotinic receptors in the PFC promoted the exploration of novel social stimuli (133). Oxytocin increased pair bonding and pro-social behavior (181–183) through contributions from sub-cortical regions and perhaps *via* their projections to the PFC (39). Social recognition memory is regulated by oxytocin-mediated modulation of prefrontal cortex plasticity, which is impaired when juvenile rats eat a high-fat diet (184). Moreover, oxytocin receptor (OTr)-expressing SST+ neurons in the murine PFC present sex-specific responses to oxytocin (185). These neurons regulate female motivation to interact with males during the estrus phase, yet do not affect interactions with other females. In another study, chronic activation of pyramidal neurons of rat PFC reduced social motivation to interact with novel stimuli in a three-chamber task (186). These motivation deficits were ameliorated by systemic OTr agonist injections. Recently, Riad et al. (187) showed that corticotrophin-releasing hormone (CRH)-expressing neurons inhibit OTr-positive neurons and layer 2/3 in the mPFC when stimulated *in vitro* at low frequency. When activated chemo-genetically, these CRH neurons increase novelty preference in male but not female mice. Moreover, a recent study showed that PFC infralimbic CRH+ neurons that project to the lateral septum modulate social novelty preference (188). In summary, more detailed studies on the effects of PFC neuromodulators are required to reveal the intricate mechanisms through which they modulate E/I balance and circuitry in this brain region and regulate its activity during specific social behaviors (Figure 3B).

## Does the PFC regulate social and affective emotional state recognition in rodents?

Social recognition and memory of socially relevant events are essential to social interactions. Early social recognition is impaired in children with ASD (189, 190). A study in rats reported that lesions in the ACC reduced social recognition, while OFC lesions did not affect this behavior (134). Activation of pyramidal neurons in *Cntnap2* knockout mice [corresponding to a pre-clinical model of cortical dysplasia focal epilepsy syndrome, a type of ASD (191)] balanced the E/I ratio and alleviated deficits in social recognition of novel juveniles (192). Mice that lack Fgf17, a signaling molecule essential for rostral forebrain development (193), show difficulties in social recognition and low c-Fos activity in the PFC during exploration of opposite sex conspecifics (194).

As discussed above, NMDA receptor hypo-function is a characteristic feature of many ASD mouse models. These deficits in glutamatergic synaptic activity also cause a loss of social recognition and memory. Moreover, acute systemic administration of the NMDA receptor antagonist MK801 reduces recognition of novel juvenile stimulus (195). Specifically, mice with NR1 subunit-deficient GABAergic neurons in the PFC do not distinguish a novel stimulus over a familiar one in a short-term social memory test (196). Collectively, PFC NMDA receptor synaptic activity contributes to social recognition and memory.

Works on empathy behavior in rats, which preferred to rescue a restrained conspecific over getting more food rewards, indicated a role of ACC projections to the Nac shell in regulating such behavior (197). Works from the Hong group (198) showed that dmPFC neuronal activity is related to the sex of the conspecific during social exploration. Furthermore, recent works exploring recognition of emotionally affected conspecifics indicated multi-faceted regulation by the PFC (122, 199). In addition, in mice fear-conditioned to avoid specific social stimuli, SST+ neurons inhibited PV+ neurons in the mPFC, thus causing disinhibition of excitatory projections from the region. These results suggest that the PFC regulates social fear conditioning or affective avoidance by increasing the excitatory drive in the circuit (200).

## Conclusion

We are rapidly enhancing our understanding of the neural mechanisms underlying social interactions. Here, we considered an ever-growing body of evidence showing that the prefrontal cortex is a hub in this process. Social interactions involve multiple processes, like decision-making, valence, and perception of the emotions of self and others. It is thus no wonder that such high-order and complex social behavior is affected by disorders like ASD and other neuropsychological comorbidities. We accordingly addressed evidence that the prefrontal circuitry is susceptible to synaptic, cellular, and molecular modifications in ASD. Such modifications bring about a myriad of social deficits, despite the majority of the current literature only reporting on deficits in sociability, social recognition, and vocalization. We suggest that studying social deficits through tasks that address affective emotions, empathic behavior, and even group dynamics will enrich our understanding of the causes of ASD in rodent models. Taken together with studies of the mechanisms and roles of various neuromodulators and transmitters in the PFC during social interactions, such explorations can better guide interventions of clinical value.

## Author contributions

AM and SW: conceptualization and writing—review and editing. SW: funding, resources, and supervision. AM: writing—original draft. All authors contributed to the article and approved the submitted version.

## Funding

This study was supported by ISF-NSFC joint research program (grant No. 3459/20 to SW), the Israel Science Foundation (grants No. 1361/17 and 2220/22 to SW), the Ministry of Science, Technology and Space of Israel (Grant No. 3-12068 to SW), the Ministry of Health of Israel (ERA-NET 2022 grant No. 3-18380), and the United States-Israel Binational Science Foundation (grant No. 2019186 to SW).

## Conflict of interest

The authors declare that the research was conducted in the absence of any commercial or financial relationships that could be construed as a potential conflict of interest.

## Publisher’s note

All claims expressed in this article are solely those of the authors and do not necessarily represent those of their affiliated organizations, or those of the publisher, the editors and the reviewers. Any product that may be evaluated in this article, or claim that may be made by its manufacturer, is not guaranteed or endorsed by the publisher.
